# Colistin Resistance Gene *mcr-1* Mediates Cell Permeability and Resistance to Hydrophobic Antibiotics

**DOI:** 10.3389/fmicb.2019.03015

**Published:** 2020-01-10

**Authors:** Baiyuan Li, Fang Yin, Xuanyu Zhao, Yunxue Guo, Weiquan Wang, Pengxia Wang, Honghui Zhu, Yeshi Yin, Xiaoxue Wang

**Affiliations:** ^1^Key Laboratory of Comprehensive Utilization of Advantage Plants Resources in Hunan South, College of Chemistry and Bioengineering, Hunan University of Science and Engineering, Yongzhou, China; ^2^Department of Breast and Thyroid Surgery, The Fifth Affiliated Hospital, Sun Yat-sen University, Zhuhai, China; ^3^Key Laboratory of Tropical Marine Bio-resources and Ecology, Guangdong Key Laboratory of Marine Materia Medica, RNAM Center for Marine Microbiology, South China Sea Institute of Oceanology, Chinese Academy of Sciences, Guangzhou, China; ^4^University of the Chinese Academy of Sciences, Beijing, China; ^5^State Key Laboratory of Applied Microbiology Southern China, Guangdong Provincial Key Laboratory of Microbial Culture Collection and Application, Guangdong Open Laboratory of Applied Microbiology, Guangdong Microbial Culture Collection Center, Guangdong Institute of Microbiology, Guangzhou, China

**Keywords:** *mcr-1*, colistin resistance, permeability, hydrophobic antibiotics, plasmid

## Abstract

Colistin is considered the last-resort antibiotic used to treat multidrug resistant bacteria-related infections. However, the discovery of the plasmid-mediated colistin resistance gene, *mcr-1*, threatens the clinical utility of colistin antibiotics. In this study, the physiological function of MCR-1, which encodes an LPS-modifying enzyme, was investigated in *E. coli* K-12. Specifically, the impact of *mcr-1* on membrane permeability and antibiotic resistance of *E*. *coli* was assessed by constructing an *mcr-1* deletion mutant and by a complementation study. The removal of the *mcr-1* gene from plasmid pHNSHP45 not only led to reduced resistance to colistin but also resulted in a significant change in the membrane permeability of *E. coli*. Unexpectedly, the removal of the *mcr-1* gene increased cell viability under high osmotic stress conditions (e.g., 7.0% NaCl) and led to increased resistance to hydrophobic antibiotics. Increased expression of *mcr-1* also resulted in decreased growth rate and changed the cellular morphology of *E. coli.* Collectively, our results revealed that the spread of *mcr-1-*carrying plasmids alters other physiological functions in addition to conferring colistin resistance.

## Introduction

Colistin is one of the primary classes of antibiotics with activity against most gram-negative bacteria and is considered the last resort antibiotic for the treatment of infections caused by carbapenem-resistant Enterobacteriaceae. In 2015, a plasmid-encoded colistin resistance gene named *mcr-1* was described in Enterobacteriaceae isolated from humans and livestock in China (Liu et al., 2016). Since then, polymyxins have rightfully drawn renewed attention to colistin resistance, and plasmid-mediated colistin resistance by *mcr-1* has been reported worldwide in livestock, food and humans (Poirel et al., 2017; Li et al., 2018). The *mcr-1* gene confers colistin resistance by encoding a phosphoethanolamine transferase that catalyzes the addition of a phosphoethanolamine moiety to lipid A in the bacterial outer membrane (OM) (Gao et al., 2016; Hinchliffe et al., 2017), which may modify the structure of lipid A and then decrease the growth rate, cell viability, and competitive ability and shape cytoplasmic structures (Yang et al., 2017).

The OM of gram-negative bacteria plays a crucial role in protecting cells against an adverse environment and exchanging material (Costerton et al., 1974). To work effectively, antibiotics must pass across the OM barricade to reach the inhibitory concentration inside the bacterial cell (Vergalli et al., 2017). Bacterial OMs with low permeability have been identified as robust barriers that prevent many antibiotics from reaching their intracellular targets (Nikaido, 2003). Antibiotics usually traverse the OM by one of two mechanisms: the lipid-mediated pathway responsible for macrolides and hydrophobic antibiotics, such as aminoglycosides (gentamycin, kanamycin), and general diffusion porins for hydrophilic antibiotics such as β-lactams (Benz, 1988; Nikaido, 2003). The lipid and protein compositions of the OM have a major impact on the susceptibility of the microorganism to antibiotics, and drug resistance involving modifications of these macromolecules is common (Benz, 1988). For instance, several studies showed that alternations in the hydrophobic properties of the membrane or null mutations in porins create resistance to β-lactam antibiotics (Miller, 2016; Ghai and Ghai, 2018). MCR-1 is a membrane-bound enzyme consisting of five hydrophobic transmembrane helixes and a soluble form located in the periplasmic space (Liu et al., 2016). A recent study reported that mutants with a high-level colistin resistance are more susceptible to most antibiotics compared with their respective parental strains (Yang et al., 2017). However, whether other antibiotic resistances could be affected by expression of *mcr-1* remain unclear. Since *mcr-1* encodes a phosphoethanolamine transferase that modifies the structure of LPS of the OM, it raises the possibility that MCR-1 may affect the susceptibility of bacteria to hydrophobic antibiotics by changing the membrane permeability. Therefore, we analyzed the impact of *mcr-1* expression on the membrane permeability of *E*. *coli* by constructing an *mcr-1* deletion mutant strain and by constructing a vector to overexpress *mcr-1*.

## Materials and Methods

### Bacterial Strains, Plasmids, and Growth Conditions

The bacterial strains and plasmids used in this study are listed in Table 1, and the sequences of primers used in this study are listed in Supplementary Table S1. The *E*. *coli* strains were grown in Luria–Bertani (LB) broth or on LB agar plates (with 10 g NaCl per liter) at 37°C, except for *E*. *coli* carrying pKD46 or pCP20, which were grown at 30°C. Antibiotics and other chemicals were used at the following final concentrations: chloramphenicol, 30 μg/ml; polymyxin B, 2 μg/ml; ampicillin, 100 μg/ml; isopropyl-β-d-thiogalactopyranoside (IPTG), 0.5 mM; and arabinose, 1 mM. A total of 1 mM L-arabinose or 0.5 mM IPTG (Sigma) was used to induce P*ara* or P*lac*, respectively.

**TABLE 1:** Strains and plasmids used in this study.

**Strains/Plasmids**	**Phenotypes**	
**Strains**		
*E. coli* K12 BW25113	*lacI*^q^ *rrnB*_T__14_ *ΔlacZ*_WJ__16_ *hsdR514 ΔaraBAD*_AH__33_ *ΔrhaBAD*_LD__78_	Baba et al. (2006)
*E. coli* C600	*F-thr-1 leuB6(Am) fhuA21 cyn-101 lacY1 glnX44(AS) λ*^–^ *e14-rfbC1 glpR200(glp^c^) thiE1*	Appleyard (1954)
Wild type	*E*. *coli* BW25113/pHNSHP45	This study
Δ*mcr-1*:*cat*	*E*. *coli* BW25113/pHNSHP45Δ*mcr-1* Cm^R^	This study
Δ*mcr-1*	*E*. *coli* BW25113/pHNSHP45Δ*mcr-1*ΔCm^R^	This study
**Plasmids**		
pHNSHP45	*mcr-1*, GenBank accession no. KP347127	Liu et al. (2016)
pKD46	Amp^R^, λ Red recombinase expression	Datsenko and Wanner (2000)
pKD3	FRT-flanked *cat* gene (Cm^R^) in *oriR*γ replicon requiring the *pir* gene product	Datsenko and Wanner (2000)
pCP20	Amp^R^ and Cm^R^; temperature-sensitive replication, thermal induction of FLP recombinase synthesis	Baba et al. (2006)
pCA24N	Cm^R^; *lacI*^q^, IPTG inducible expression vector in *E. coli*	Kitagawa et al. (2005)
pCA24N-*mcr-1*	Cm^R^; *lacI*^q^, P_T__5__–lac_:*mcr-1*	This study
pCA24N-*mcr-1*-*gfp*	Cm^R^; *lacI*^q^, P_T__5__–lac_:*mcr-1-gfp*	This study

### Construction of the Deletion Mutant

The coding region of the *mcr-1* gene was deleted from *E. coli* K12 BW25113 carrying plasmid pHNSHP45 (Liu et al., 2016) following a one-step inactivation method (Datsenko and Wanner, 2000). The primers used in this study are shown in Supplementary Table S1. PCR products containing chloramphenicol resistance cassettes flanked by 39 bp of homology to the 5′ and 3′ termini of *mcr-1* were electroporated into competent cells of parent strains carrying pKD46. To construct the *mcr-1* deletion mutant, PCR products that included 37-nt homology extensions and 20-nt priming sequences for the chloramphenicol resistance gene *cat*, bordered by FLP recombination target (FRT) sites, were amplified from plasmid pKD3 (*cat*) using primers pKD46-mcrF/pKD46-mcrR. Removal of the *mcr-1* gene from pHNSHP45 in *E. coli* was verified by PCR and DNA sequencing using the primer pair MCR-LF/MCR-LR. The FRT-flanked chloramphenicol cassette was removed after transformation with pCP20 as described previously (Datsenko and Wanner, 2000). pCP20 is a plasmid that carries ampicillin and chloramphenicol resistance genes and exhibits temperature-sensitive replication and thermal induction of FLP synthesis (Cherepanov and Wackernagel, 1995). CmR mutants were transformed with pCP20, and ampicillin-resistant transformants were selected at 30°C, propagated non-selectively at 42°C and then tested for loss of all antibiotic resistances.

### Plasmid Constructs

The pCA24N vector was used to express target genes in *E. coli*. The coding region of *mcr-1* was PCR-amplified from genomic DNA of *E. coli* carrying pHNSHP45 using the primer pair pCA24N-mcrF/pCA24N-mcrR. PCR products were digested with *Sal*I and *Xba*I and inserted into the corresponding sites of pCA24N. The correct constructs were verified by PCR with primer pair pCA24N-F/pCA24N-R and DNA sequencing. The same procedures were performed to fuse *gfp* before the stop codon of the *mcr-1* gene, as well as for the construction of the plasmid pCA24N-*mcr-1-gfp*. To generate pCA24N-*mcr-1-gfp*, the coding region of *mcr-1* without its stop codon was amplified with primers Mcr-1F(salI) and Mcr-1R(xbaI), and the coding region of *gfp* was amplified using pCA24N-*gfp* (Guo et al., 2017) as the template with primers Gfp-F(xbaI) and Gfp-R(*Eco*RI). The *mcr-1* fragment was digested with *Sal*I and *Xba*I, the *gfp* fragment was digested *Xba*I and RcoRI, and inserted into the corresponding sites of pCA24N. pCA24N-based expression vectors were transferred into the *E. coli* BW25113 host.

### Antibiotics Susceptibility Testing

The antibiotics tested include ampicillin, polymyxin B (PB), ceftazidime, ciprofloxacin, gentamycin (GEN), kanamycin, chloramphenicol, tetracycline, rifampicin, nalidixic acid, and spectinomycin. The antibiotic resistance level was described by the minimum inhibitory concentrations (MICs) determined using a custom-made 96-well MIC panel (Flentie et al., 2019). The results were interpreted according to the criteria of the Clinical and Laboratory Standards Institute (CLSI) (Clinical and Laboratory Standards Institute, 2017).

### Microscopy Exam

To evaluate cell membrane integrity, the membrane-specific red-fluorescent dye FM4-64 (Thermo Fisher Scientific, Rockford, IL, United States) was used. Overnight cultures were diluted in 50 ml of fresh medium and cultured to an OD_600 nm_ of 0.5, and cells were harvested by centrifugation (6000 × *g*, 2 min), washed and resuspended in phosphate buffered saline (PBS). Cells were then treated with 0.85% NaCl or 7.0% NaCl for 30 min followed by staining with 4 μg/ml FM 4-64 for 15 min in the dark at ambient temperature. Bacterial cells were imaged using a Zeiss Axiovert fluorescence microscope (Carl Zeiss Inc., Thornwood, NY, United States).

### Protein Localization

For localization of MCR-1 by GFP fusion, overnight cultures of BW25113 carrying pCA24N-*mcr-1*-*gfp* were inoculated into LB broth supplemented with chloramphenicol (30 μg/ml) to an OD_600 nm_ of 0.1 and 0.5 mM IPTG was added to induce MCR-1-GFP expression for 2 h before imaging. Cells were washed with PBS and imaged with fluorescence microscopy (Zeiss Axiophot) using an oil immersion objective (100×).

## Results and Discussion

### The Expression of *mcr-1* Decreases Resistance to Hydrophobic Antibiotics

**FIGURE 1:**
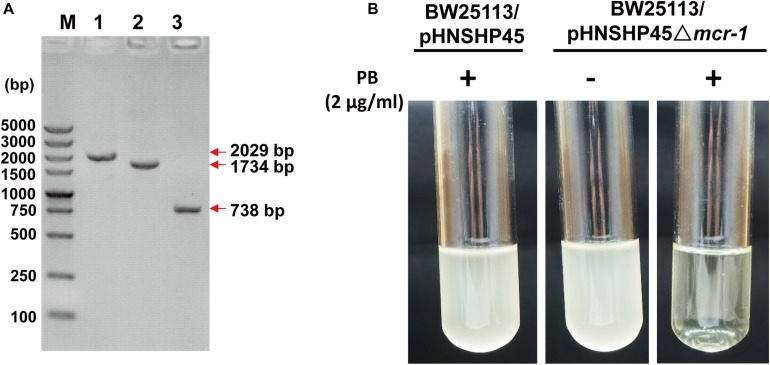
Confirmation of the deletion of *mcr-1* from *E*. *coli* K12 BW25113/pHNSHP45. **(A)** PCR detection of *mcr-1* deletion mutants using the *mcr-1* flanking primer pair MCR-LF/LR. M, DNA marker DS5000. 1, the wild-type strain (*E*. *coli* K12 BW25113/pHNSHP45); 2, the deletion mutant Δ*mcr-1*:*cat* (*E*. *coli* K12 BW25113/pHNSHP45 Δ*mcr-1*:*cat*); 3, the deletion mutant Δ*mcr-1* (*E*. *coli* K12 BW25113/pHNSHP45 Δ*mcr-1*). **(B)** The polymyxin B (PB) susceptibility test of the wild-type strain and the Δ*mcr-1* strain.

The plasmid pHNSHP45 was extracted from *E*. *coli* K12 C600/pHNSHP45 using the EZNA^®^ plasmid mini kit I (Omega) and then electroporated into the *E*. *coli* K12 BW25113 host, which is susceptible to polymyxin B (MIC = 0.5 μg/mL). Positive clones were selected with 2 μg/mL polymyxin B and confirmed by PCR with the primer pair CLR5-F/R (Supplementary Table S1). To explore the impact of *mcr-1*, we deleted the *mcr*-1 gene with a one-step inactivated method using a λ-red recombinase expression plasmid pKD46 as described by Datsenko and Wanner (2000). The replacement of the *mcr-1* gene with the *cat* cassette amplified from pKD3 (strain BW25113/pHNSHP45 Δ*mcr-1*:*cat*) and the elimination of the FRT-flanked *cat* cassette by using the FLP recombinase expression plasmid pCP20 to obtain strain BW25113/pHNSHP45 Δ*mcr-1* were confirmed by PCR and DNA sequencing (Figure 1). As expected, the deletion of *mcr-1* resulted in decreased resistance to polymyxin B (MIC = 0.5 μg/mL) (Figure 1B). The MIC value of PB for the *mcr-1* deletion mutant was 0.5 μg/ml compared to an 8 μg/ml for the wild type (Table 2).

**TABLE 2:** Antimicrobial susceptibility of *E*. *coli* BW25113, the wild-type strain and the *mcr-1* deletion mutant.

**Antibiotics**	**MIC (μg/mL)**		
	**BW25113**	**Wild type**	**Δ*mcr-1***
Polymyxin B	0.5	8	0.5
Gentamicin	16	8	16
Kanamycin	8	4	8
Rifampicin	16	8	16
Ampicillin	4	4	4
Ciprofloxacin	0.125	0.125	0.125
Ceftazidime	0.5	0.5	0.5
Chloramphenicol	8	8	8
Tetracycline	4	4	4
Nalidixic acid	4	4	4
Spectinomycin	32	32	32

*MIC values (μg/mL) of 11 antibiotics are listed. Three independent cultures of each strain were tested.*

The asymmetric lipopolysaccharide (LPS)-phospholipid bilayer of the OM provides a formidable permeability barrier for both hydrophilic and hydrophobic antibiotics (Nikaido, 2003; Zgurskaya et al., 2015). Previous studies have found that small hydrophilic drugs use the pore-forming porins to cross the OM, while hydrophobic drugs diffuse across the LPS-phospholipid bilayer (Vaara, 1992; Nikaido, 2003). To explore whether MCR-1 expression demonstrates differences in different families of antibiotics, we performed MIC tests of the Δ*mcr-1* and wild-type strains using a 96-well MIC panel test assay that contained 11 antibiotics. As expected, the Δ*mcr-1* strain showed increased resistance to gentamicin, kanamycin and rifampicin. However, no effect was found for resistance to ampicillin, nalidixic acid, spectinomycin, or ciprofloxacin (Table 2). Interestingly, gentamicin, kanamycin, and rifampicin are hydrophobic antibiotics, while ampicillin, nalidixic acid, spectinomycin, and ciprofloxacin are hydrophilic antibiotics. Thus, the deletion of *mcr-1* also increases resistance to hydrophilic antibiotics. *mcr-1* confers colistin resistance through the addition of cationic phosphoethanolamine (pEtN) to phosphate groups on the lipid A component of LPS, which reduces the net anionic charge of the cell surface (Jeannot et al., 2017). LPS modification in Gram-negative bacteria plays a significant role in resistance to antimicrobial factors (Gunn, 2001). Thus, we proposed that the expression of *mcr-1* resulted in the modification of LPS and disrupt the organization of the LPS-phospholipid bilayer, change its permeability, and therefore decrease the resistance to hydrophobic antibiotics, but the hydrophilic antibiotics traverse the OM through porin channels.

### The Expression of *mcr-1* Decreases Survival During High Salt Stress

**FIGURE 2:**
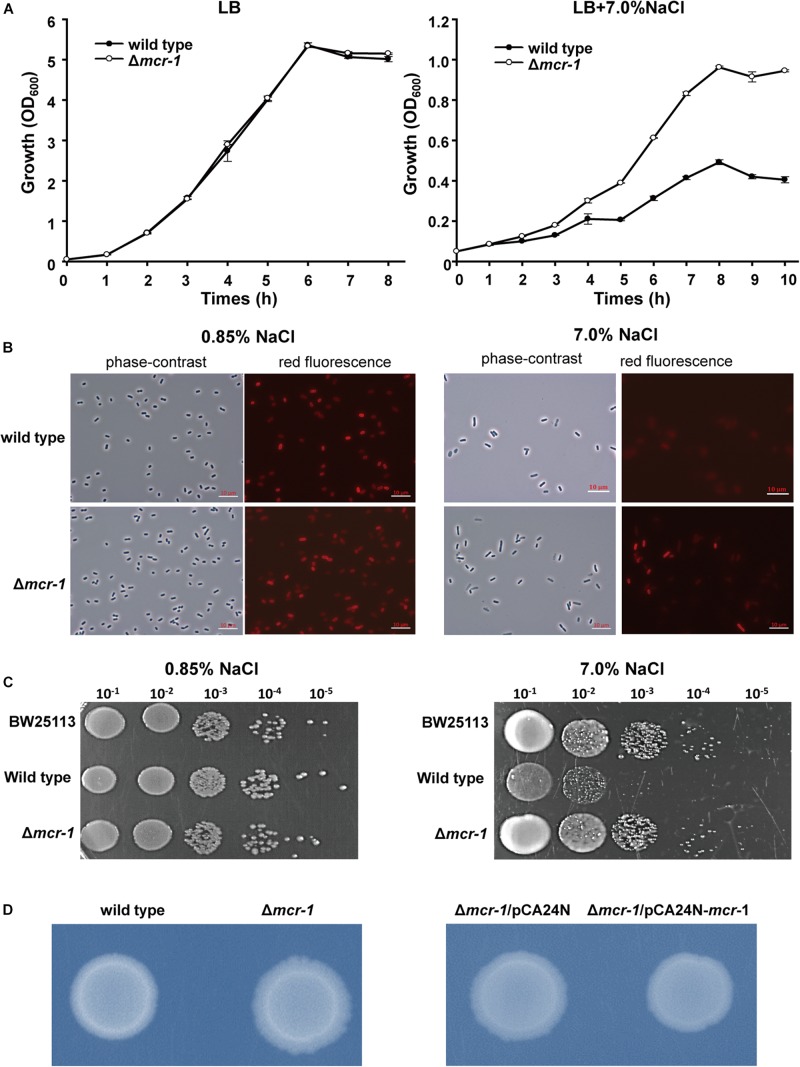
The effect of *mcr-1* expression on the osmotic tolerance of *E*. *coli*. **(A)** Growth of the two strains in LB broth containing 1% NaCl or LB broth containing 7% NaCl. **(B)** Phase-contrast microscopy and fluorescence images of Δ*mcr-1* and wild-type cells. Exponentially growing cells (OD_600 nm_∼ 0.5) were taken after 30 min of incubation with LB broth containing 0.85% NaCl or 7.0% NaCl. **(C)** Cell viability of exponentially growing cells (OD_600 nm_∼0.5) after 30 min of incubation with LB broth containing 0.85% NaCl or 7.0% NaCl. Experiments were conducted with three independent cultures, and only representative images are shown in panels **(B,C)**. **(D)** Colony morphology of *E. coli* with or without *mcr-1* on LB agar plates.

Acquired antibiotic resistance by horizontal gene transfer tends to be related to a fitness cost for bacterial hosts (Andersson and Levin, 1999; Andersson and Hughes, 2010; Vogwill and MacLean, 2015). The osmotic stresses of *E*. *coli* strains with and without *mcr-1* were tested. The growth of the wild type strain was severely hindered in the presence of 7.0% NaCl in LB broth (total NaCl concentration was 7.0%) when compared with the Δ*mcr-1* strain, while no growth defect was observed for the two strains in regular LB broth containing 1.0% NaCl (Figure 2A). In addition, the growth of *E*. *coli* with or without *mcr-1* in LB broth with different NaCl concentration was tested and the cell density was measured by optical density at 600 nm. The results showed that the cell density of the *E*. *coli* was decreased with the increase of NaCl concentration, and *E*. *coli* without *mcr-1* was able to tolerance to higher NaCl concentration than *E*. *coli* with *mcr-1* (Supplementary Table S2). Furthermore, we examined cell integrity using the red membrane dye FM 4-64 (Life Technologies, United States), which specifically stains the cell membrane. Exponentially growing cells (OD_600 nm_∼ 0.5) were collected, washed and incubated with 0.85% NaCl or 7.0% NaCl for 30 min. Cells were then collected and imaged by fluorescence microscopy (Zeiss Axiophot) using an oil immersion objective (100×). The cell membranes appeared intact in the presence of low concentrations of NaCl, while membrane integrity of wild type strain was only severely reduced in the presence of 7% NaCl (The membrane defects of wild type strain was about 79.2% compared with Δ*mcr-1* strain is 19.4%) (Figure 2B). Consistent with the above results, the Δ*mcr-1* strain demonstrated 10-fold higher viability than the wild-type cells in the presence of 7% NaCl (Figure 2C). Taken together, these results showed that the expression of the *mcr-1* gene decreases tolerance to high salt stress. We proposed that the mechanism of the expression of the *mcr-1* gene decreases cell fitness under high salt conditions may be due to expression of the *mcr-1* gene affects the membrane permeability.

### The Expression of *mcr-1* Affects the Growth and Cell Morphology of *E*. *coli*

**FIGURE 3:**
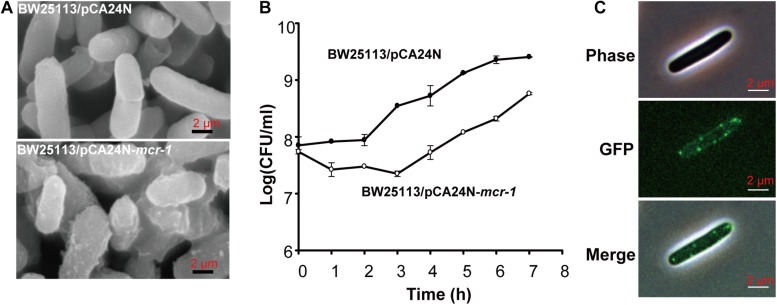
The effect of *mcr-1* expression on the morphology of *E*. *coli*. **(A)** SEM of *E*. *coli* K-12 BW25113 carrying empty plasmid or pCA24N-*mcr-1*. **(B)** Cell viability of *E*. *coli* K-12 BW25113 carrying empty plasmid or pCA24N-*mcr-1*. When the cell density reached to OD_600 nm_ of 0.5, IPTG was added to the final concentration of 0.5 mM and the growth curve was established by monitoring cell density (CFU/mL) every 1 h via plate count method. Three independent cultures of each strain were tested. **(C)** Localization of the fused protein MCR-1-GFP. BW25113 carrying pCA24N-*mcr-1-gfp* was induced by 0.5 mM IPTG for 2 h. Cells were examined by phase contrast microscopy (upper panel) and fluorescence microscopy (middle panel). At least two independent cultures were tested and only representative images are shown in panels **(A,C)**.

After prolonged incubation on LB agar plates for 24 h, the edge of the colonies formed by the Δ*mcr-1* strain appeared more wrinkle compared with the wild-type strain (Figure 2D). Furthermore, the *mcr-1* coding region from pHNSHP45 was cloned into plasmid pCA24N to construct pCA24N-*mcr-1*. To further check the cell morphology of *E*. *coli*, the complementation experiments were carried out. As expected, the edge of the colonies formed by the Δ*mcr-1*/pCA24N strain appeared more wrinkle compared with the Δ*mcr-1*/pCA24N-*mcr-1* (Figure 2D). Scanning electron microscopy (SEM) was then employed to study the cell morphology of *E*. *coli* K-12 BW25113/pCA24N-*mcr-1* with the addition of 0.5 mM IPTG. As shown in Figure 3A, cells overexpressing *mcr-1* via pCA24N-*mcr-1* had rougher cell envelopes than the cells carrying empty pCA24N. In addition, overexpression of *mcr-1* significantly reduced the cell growth rate (Figure 3B), which is consistent with a recent report indicating that increased expression of *mcr-1* results in decreased growth rate and cell viability (Yang et al., 2017). To further check the localization of MCR-1 in *E*. *coli*, the green fluorescence protein gene *gfp* was fused to the C-terminus of the *mcr-1* gene to express the fused protein MCR-1-GFP. As expected, MCR-1-GFP was located around the cellular membrane (Figure 3C), which further confirmed that MCR-1 is a membrane protein. As a control, the green fluorescence protein GFP was produced using pCA24N-*gfp* and the protein GFP was localized to the cytoplasm (Supplementary Figure S1). To further determine if the MCR-1-GFP fusion protein confers polymyxin resistance, we performed MIC tests of the BW25113/pCA24N and BW25113/pCA24N-*mcr-1*-*gfp* strains using a 96-well MIC panel test assay. The MIC value of PB for the BW25113/pCA24N-*mcr-1*-*gfp* was 8.0 μg/ml, but BW25113/pCA24N was 0.5 μg/ml, suggesting that GFP fused to the MCR-1 does not affect the polymyxin resistance of MCR-1. Thus, MCR-1 is localized at the membrane, and the expression of *mcr-1* affects cellular morphology and colony morphology.

## Conclusion

In this study, we demonstrated that MCR-1 is a membrane protein that localizes to the cellular membrane. Furthermore, MCR-1 increases the loss of the cell membrane integrity and decreases the MICs of gentamicin, kanamycin and rifampicin. Evolving colistin resistance by acquiring *mcr-1* therefore challenges bacterial populations with an evolutionary trade-off: input of *mcr-1* protects the host against colistin but changes the membrane permeability and reduces resistance to hydrophobic antibiotics. This trade-off may further explain the balance between *mcr-1* expression and bacterial survival. Several studies have documented a link between antibiotic use and the development of antibiotic resistance (Goossens et al., 2005; Bergman et al., 2009). Our results provide a further possibility of *mcr-1* gene transfer, which was affected not only by colistin use but also by environmental cues, such as osmotic pressure conditions.

## Data Availability Statement

All datasets generated for this study are included in the article/Supplementary Material.

## Author Contributions

XW and BL conceptualized and designed the project. BL, XW, FY, XZ, YG, WW, HZ, PW, and YY did the investigation, and data curation and analysis. BL and XW did the supervision and visualization. BL and XW wrote, reviewed, and edited the original draft.

## Conflict of Interest

The authors declare that the research was conducted in the absence of any commercial or financial relationships that could be construed as a potential conflict of interest.
