# Supplementary material for: Colistin Resistance Gene mcr-1 Mediates Cell Permeability and Resistance to Hydrophobic Antibiotics
Source: Front Microbiol. 2020 Jan 10;10:3015. doi: 10.3389/fmicb.2019.03015 (PMC6966882; doi:10.3389/fmicb.2019.03015)

**supplementary materiaL**

**Colistin Resistance Gene *mcr-1* Mediates Cell Permeability and Resistance to Hydrophobic Antibiotics**

Baiyuan Li^1^, Fang Yin^2^, Xuanyu Zhao^3,4^, Yunxue Guo^3^, Weiquan Wang^3,4^, Pengxia Wang^3^, Honghui Zhu^5^, Yeshi Yin^1^, Xiaoxue Wang^3,4*^

^1^Key Laboratory of Comprehensive Utilization of Advantage Plants Resources in Hunan South, College of Chemistry and Bioengineering, Hunan University of Science and Engineering, Yongzhou, Hunan, China

^2^Department of Breast and Thyroid Surgery，the 5th Affiliated Hospital, Sun Yat-sen University, Zhu Hai, 519000，China

^3^Key Laboratory of Tropical Marine Bio-resources and Ecology, Guangdong Key Laboratory of Marine Materia Medica, RNAM Center for Marine Microbiology, South China Sea Institute of Oceanology, Chinese Academy of Sciences, Guangzhou 510301, PR China

^4^University of Chinese Academy of Sciences, Beijing 100049, China

^5^State Key Laboratory of Applied Microbiology Southern China, Guangdong Provincial Key Laboratory of Microbial Culture Collection and Application, Guangdong Microbial Culture Collection Center (GDMCC), Guangdong Open Laboratory of Applied Microbiology, Guangdong Institute of Microbiology, Guangzhou, China

^*^Correspondence and requests for materials should be addressed to: [xxwang@scsio.ac.cn](mailto:xxwang@scsio.ac.cn)

**Keywords**: *mcr-1*, colistin resistance, permeability, hydrophobic antibiotics, plasmid

**Supplementary Table S1** primers used in this study.

| Primers | Sequence (5’-3’) |
| --- | --- |
| CLR5-F | CGGTCAGTCCGTTTGTTC |
| CLR5-R | CTTGGTCGGTCTGTAGGG |
| pKD46-mcrF | GTAATTATCCCACCGTTTATTTTTTGAGTAGTTTCTCGTGTAGGCTGGAGCTGCTTC |
| pKD46-mcrR | GCAGCCACTGGATACTTTGAGCGATGAAATCATCGGTTCCATATGAATATCCTCCTT |
| MCR-LF | CATCAATCAGTGGAGCGAAG |
| MCR-LR | AATACGGCATAACAAACCCC |
| pCA24N-mcrF | ACGCGTCGACATGCAGCATACTTCTGTGTGGT |
| pCA24N-mcrR | GCTCTAGATCAGCGGATGAATGCGGTGCGG |
| Mcr-1F(salI) | GCGTCGACATGATGCAGCATACTTCTGTGTGGT |
| Mcr-1R(xbaI) | GCTCTAGAGCGGATGAATGCGGTGCGGTCT |
| Gfp-F(xbaI) | GCTCTAGAATGCTATGCGGCCGCAGTAAAG |
| Gfp-R(EcoRI) | CCGGAATTCTTATTTGTATAGTTCATCCATGC |
| pCA24N-F | GATAACAATTTCACACAGAATT |
| pCA24N-R | GTCAGAGGTTTTCACCGTCATCA |

**Supplementary Table S2** Growth of *E*. *coli* with or without *mcr-1* in LB broth with different NaCl concentrations. The OD_600_ was measured after 24 h incubation (37 °C).

| NaCl  concentrations | OD_600_ | | | |
| --- | --- | --- | --- | --- |
|  | Wild type | Δ*mcr-1* | BW25113/pCA24N | BW25113/pCA24N-*mcr-1* |
| 1% | 0.612 | 0.623 | 0.649 | 0.583 |
| 2% | 0.536 | 0.570 | 0.524 | 0.449 |
| 3% | 0.461 | 0.471 | 0.476 | 0.348 |
| 4% | 0.231 | 0.300 | 0.329 | 0.302 |
| 5% | 0.183 | 0.195 | 0.242 | **0.245** |
| 6% | 0.191 | 0.209 | 0.216 | 0.054 |
| 7% | **0.174** | 0.216 | 0.228 | 0.081 |
| 8% | 0.08 | **0.207** | **0.110** | 0.062 |
| 9% | 0.056 | 0.056 | 0.055 | 0.053 |

**Supplementary** Figure S1. Localization of the protein GFP. BW25113 carrying pCA24N- *gfp* was induced by 0.5 mM IPTG for 2 h.


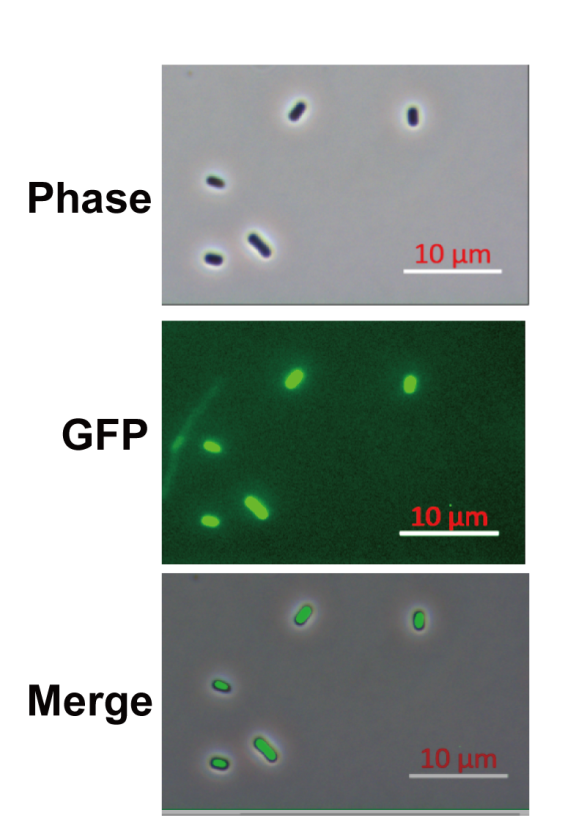

Supplement: Supplementary file 1 [file Data_Sheet_1.docx]
